# Full-term extrauterine abdominal pregnancy: a case report

**DOI:** 10.1186/1752-1947-5-531

**Published:** 2011-10-31

**Authors:** Amal A Dahab, Rahma Aburass, Wasima Shawkat, Reem Babgi, Ola Essa, Razaz H Mujallid

**Affiliations:** 1 Department of Obstetrics and Gynecology, Maternity and Children Hospital, Jeddah, Saudi Arabia; 2 Department of Surgery, Maternity and Children Hospital, Jeddah, Saudi Arabia; 3 Department of Anesthesia, Maternity and Children Hospital, Jeddah, Saudi Arabia

## Abstract

**Introduction:**

Extrauterine abdominal pregnancy is extremely rare and is frequently missed during antenatal care. This is a report of a full-term extrauterine abdominal pregnancy in a primigravida who likely had a ruptured ectopic pregnancy with secondary implantation and subsequently delivered a healthy baby.

**Case presentation:**

A 23-year-old, Middle Eastern, primigravida presented at 14 weeks gestation with intermittent suprapubic pain and dysuria. An abdominal ultrasound examination showed a single viable fetus with free fluid in her abdomen. A follow-up examination at term showed a breech presentation and the possibility of a bicornute uterus with the fetus present in the left horn of her uterus. Our patient underwent Cesarean delivery under general anesthesia and was found to have a small intact uterus with the fetus lying in her abdomen and surrounded by an amniotic fluid-filled sac. The baby was extracted uneventfully, but the placenta was implanted in the left broad ligament and its removal resulted in massive intraoperative bleeding that necessitated blood and blood products transfusion and the administration of Factor VII to control the bleeding. Both the mother and newborn were discharged home in good condition.

**Conclusions:**

An extrauterine abdominal pregnancy secondary to a ruptured ectopic pregnancy with secondary implantation could be missed during antenatal care and continue to term with good maternal and fetal outcome. An advanced extrauterine pregnancy should not result in the automatic termination of the pregnancy.

## Introduction

An extrauterine abdominal pregnancy is a very rare form of ectopic pregnancy where implantation occurs within the peritoneal cavity, outside the Fallopian tube and ovary. It is estimated to occur in 10 out of 100,000 pregnancies in the United States [1]. The diagnosis of such a condition is frequently missed during antenatal care, despite the routine use of abdominal ultrasonography. However, it is extremely important to detect an extrauterine abdominal pregnancy because the associated maternal mortality rate is estimated at about five per 1000 cases, which is approximately seven times higher than the estimated rate for ectopic pregnancy in general, and about 90 times the maternal mortality rate associated with normal delivery in the United States [1]. Survival of the newborn is also affected with a perinatal mortality rate of 40% to 95% [2]. We report on a successful operative delivery of a healthy baby following a full-term extrauterine abdominal pregnancy in a primigravida in whom the diagnosis was missed despite repeated ultrasonography during the antenatal period.

## Case presentation

**Figure 1:**
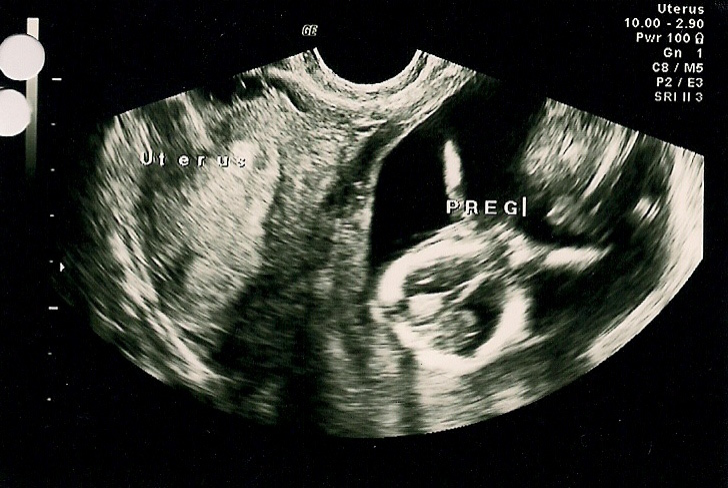
**Ultrasonography picture at 14 weeks gestation showing a single fetus, corresponding to date in size, and the possibility of a bicornute uterus**.

**Figure 2:**
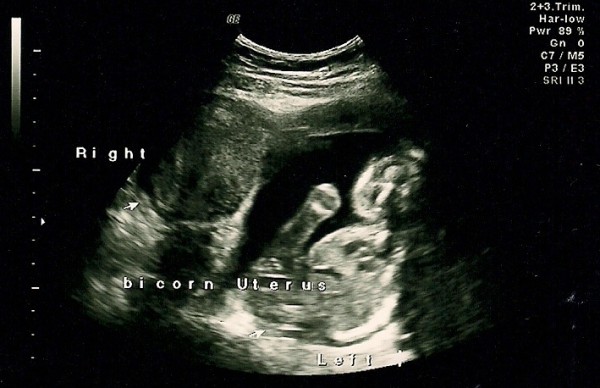
**Ultrasonography picture at 19 weeks showing fetus, amniotic fluid and the possibility of a bicornute uterus**.

**Figure 3:**
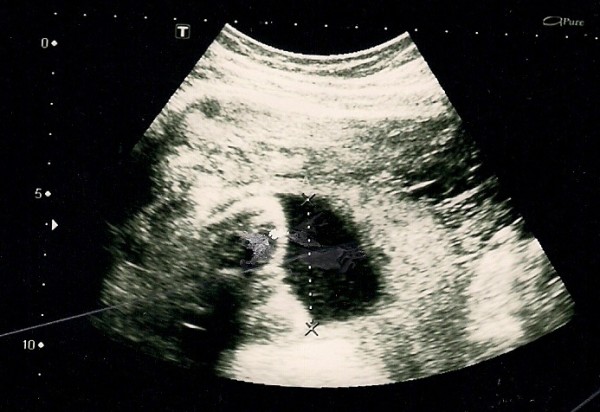
**Ultrasonography picture at 23 weeks showing fetus, amniotic fluid and normal fetal morphology**.

A 23-year-old, Middle Eastern primigravida presented to our Emergency Department at 14 weeks gestation with a two-week history of intermittent suprapubic pain associated with dysuria. On examination, she had a heart rate of 102 beats/min, her blood pressure was 109/71 mmHg, a respiratory rate of 15 breaths/min and temperature of 37.4°C. Examination of her cardiac and respiratory systems was unremarkable. Her abdomen was soft, but with mild suprapubic tenderness. Her laboratory results showed a hemoglobin level of 7.9 g/dL, hematocrit 25.7%, white blood cells 9700 cells/mm^3^, platelets 367 cells/mm^3^, serum urea 14.8 mmol/L and serum creatinine 47 μmol/L. Her serum electrolytes, coagulation profile and liver function tests were all within normal limits. Her serum β-human chorionic gonadotropin level was 75,542 IU. A bedside urine analysis showed pus cells and a urine culture subsequently grew *Streptococcus agalactiae*, which was sensitive to penicillin and amoxicillin. An ultrasound examination in our Emergency Room showed a single viable fetus with a crown-rump length corresponding to 13 weeks and five days gestation, the anterior placenta and a normal amount of liquor. A significant amount of localized fluid in the left side of her abdomen was also noted and was thought to be either ascites or blood. Our patient received intravenous amoxicillin/clavulanic acid (1 g) and 500 mL of normal saline; her pain subsided, and she was admitted to the ward for follow-up and further investigation. Iron deficiency anemia was diagnosed based on a negative sickle cell test, normal hemoglobin electrophoresis, a serum iron level of 32 μg/dL, serum ferritin of 89.7 μg/dL and a total iron binding capacity of 117 μg/dL. Our patient was placed on iron supplements. Four days later, repeat abdominal ultrasound examination suggested the presence of a bicornute uterus with the fetus in the left horn, and free fluid was noted in her pelvis (Figure 1). Her liver, spleen, kidneys and urinary bladder appeared normal. A speculum examination indicated the presence of a single cervix. An abdominal fluid tap was offered to our patient but she declined and she was discharged home on iron supplements and requested to attend outpatient follow-up. At 20 weeks gestation, our patient's hemoglobin was 9.5 g/dL and a follow-up abdominal ultrasound examination performed by a more experienced radiologist showed similar findings to the previous examination with a vertical pocket of amniotic fluid that measured 4.2 cm (Figures 2 and 3). At 40 weeks gestation, a follow-up ultrasound examination showed breech presentation with a highly vascular placenta. An external cephalic version was offered to our patient but she declined. She was admitted to the hospital for an elective Cesarean delivery. She opted for general anesthesia which was induced with propofol and suxamethonium chloride, and was maintained with sevoflurane and an oxygen/air mixture. A Pfannenstiel incision was made and her uterus was found to be intact and small on entering her abdomen. The fetus was found in her abdomen surrounded by an amniotic membrane filled with liquor. The amniotic membrane was dissected and incised and the fetus was extracted (see Additional file 1: Movie 1 showing delivery of the baby). The fetal Apgar scores were 6 and 10 at one and five minutes, respectively. The placenta was attached to the posterior aspect of the left broad ligament. During its removal, massive bleeding from the placental bed occurred and our patient became hypotensive. She was aggressively resuscitated with a total of 4000 mL of Ringer's lactate, 7 units of packed red blood cells, 4 units of fresh frozen plasma, 10 units of cryoprecipitate and 2 units of platelets. She continued to bleed and was administered 90 units/kg of intravenous Factor VII, which controlled her bleeding. Her left ovary and tube were found to be distorted while the right ones were normal. A hemostatic suture was applied on the distorted tube which was left, together with the ovary, *in situ*. An abdominal drain was inserted and our patient was extubated on the table and transferred to our Intensive Care Unit for monitoring. She was discharged to the ward on the following day and went home with her newborn 10 days after surgery.

## Discussion

Extrauterine abdominal pregnancy beyond 20 weeks gestation and with a viable fetus is a rare condition, with an estimated prevalence of one out of 8099 hospital deliveries [3], and is classified into two types. Primary abdominal pregnancy refers to pregnancy where implantation of the fertilized ovum occurs directly in the abdominal cavity. In such cases, the Fallopian tubes and ovaries are intact. There were only 24 cases of primary abdominal pregnancy reported up to 2007 [4]. In contrast, secondary abdominal pregnancy accounts for most cases of advanced extrauterine pregnancy. It occurs following an extrauterine tubal pregnancy that ruptures and gets re-implanted within the abdomen [5]. Under these circumstances, there is evidence of tubal or ovarian damage.

In this report, the intermittent suprapubic pain that our patient experienced early in her pregnancy, the free fluid seen on ultrasound examination, and the intraoperative findings of a severely distorted left Fallopian tube and ovary are highly suggestive of a tubal pregnancy that ruptured and resulted in secondary implantation in the broad ligament. Accordingly, this was most likely a case of secondary abdominal pregnancy. The diagnosis was unfortunately missed during antenatal care, and the ultrasound examination findings were repeatedly misinterpreted as an intrauterine pregnancy in a bicornute uterus. A recent report of 163 cases of extrauterine abdominal pregnancy demonstrated that the diagnosis of this condition is frequently missed, with only about 45% of cases diagnosed during the antenatal period [3]. The fact that our patient's low hemoglobin was explained by the presence of iron deficiency, her suprapubic pain was attributed to a urinary tract infection and that the free fluid in her abdomen was thought to be ascites collectively contributed to the failure to consider the possibility of an extrauterine pregnancy. Had this been discovered at an earlier stage, our patient could have been admitted to hospital for closer monitoring and her operative delivery would have been performed at an earlier gestational age.

It is interesting to note that patients with an extrauterine abdominal pregnancy typically have persistent abdominal and/or gastrointestinal symptoms during their pregnancy [5]. Our patient, however, did not have any symptoms during her pregnancy other than the intermittent suprapubic pain that she experienced at the end of her first trimester.

Extrauterine abdominal pregnancy is typically suspected when the baby's parts are easily felt on clinical examination or when the baby's lie is abnormal [6]. In our current patient, the baby was always in the breech position and the abdominal examination was always reported as being unremarkable. This could be attributed, at least in part, to the fact that our patient was examined by different physicians during her antenatal visits and the attending physician only reviewed her records. The amniotic fluid around the baby could have also contributed to the difficulty in feeling the baby's parts on abdominal examination. Ultrasonography, however, remains the main method for the diagnosis of extrauterine pregnancy. It usually shows no uterine wall surrounding the fetus, fetal parts that are very close to the abdominal wall, abnormal lie and/or no amniotic fluid between the placenta and the fetus [6]. Interestingly, amniotic fluid was detected in all ultrasound examinations in this patient but it was technically difficult to estimate its amount. The impression that the patient had a bicornute uterus was likely due to the fact that the fetus was lying behind the uterus and the empty uterine cavity was mistaken for the empty horn. Magnetic resonance imaging and serum α-fetoprotein have been used to diagnose abdominal pregnancy [4,7], however, there was no justification to perform these tests in this patient as the diagnosis was not suspected.

About 21% of babies born after an extrauterine abdominal pregnancy have birth defects, presumably due to compression of the fetus in the absence of the amniotic fluid buffer. Typical deformities include limb defects, facial and cranial asymmetry, joint abnormalities and central nervous malformation [8]. In this case, the baby was protected by the surrounding amniotic fluid and sac which could explain the absence of deformities in the baby.

The massive bleeding that occurred when the placenta was removed was due to the adherence of the placenta to the broad ligament which, unlike the uterus, does not contract. It has been reported that, unless the placenta can be easily tied off or removed, it may be preferable to leave it in place and allow for its natural regression [5,6]. However, leaving the placenta *in situ *has been associated with increased postoperative morbidity and mortality [9] and is thus not advisable. There have been many reports of advanced extrauterine pregnancy that ended with a viable fetus and a healthy mother [3]. Since the diagnosis is frequently missed preoperatively [3] and adverse fetal and maternal outcome does not necessarily occur in association with the continuation of pregnancy, one could argue that the termination of an advanced extrauterine pregnancy upon antenatal diagnosis might not be warranted. However, these cases should be followed-up closely when the diagnosis is made to prevent adverse outcomes.

## Conclusion

This is a report of an extrauterine abdominal pregnancy that had likely originated in the left Fallopian tube which ruptured and resulted in secondary implantation in the broad ligament. The pregnancy continued uneventfully to full term and ended successfully with operative delivery of a healthy baby. The importance of this case report is the fact that an extrauterine abdominal pregnancy could be missed during antenatal care despite repeated ultrasound examinations. Furthermore, the antenatal diagnosis of advanced extrauterine pregnancy does not necessarily justify the termination of the pregnancy since good maternal and fetal outcome is not uncommon.

## Consent

Written informed consent was obtained from the patient for publication of this case report and the accompanying images and video. A copy of the written consent is available for review by the Editor-in-Chief of this journal.

## Competing interests

The authors declare that they have no competing interests.

## Authors' contributions

AAD, RB and OE performed the Cesarean delivery and followed up the patient and baby postoperatively until discharge from the hospital. WS helped during the surgery from a general surgical stand point. RA was the consultant who followed up the patient during antenatal care and performed the ultrasound examinations. RHM provided the perioperative anesthetic care for the patient and was a major contributor in writing the manuscript. All authors read and approved the final manuscript.

## Supplementary Material

Additional file 1 **Cesarean delivery**. Movie file showing Cesarean delivery of the baby. Click here for file
